# Between alternative and traditional social platforms: the case of gab in exploring the narratives on the pandemic and vaccines

**DOI:** 10.3389/fsoc.2023.1143263

**Published:** 2023-07-17

**Authors:** Suania Acampa, Noemi Crescentini, Giuseppe Michele Padricelli

**Affiliations:** Department of Social Sciences, University of Naples Federico II, Naples, Italy

**Keywords:** deplatforming, alternative platform, content analysis, COVID and vaccines, pandemic reactions

## Abstract

The phenomenon of deplatforming intended as the removal of social media accounts because of breaking rules on mainstream platforms such as Facebook, Twitter, YouTube, and Instagram recently increased due to new terms and conditions of use of digital media, and new alternative social media platforms emerged and presented themselves as protectors of freedom expression. In this way, it becomes interesting to understand better the context of these platforms' so-called *web suburbs* that consist in those digital places that ≪host what we can generally call “subcultures,” including fandoms, religious sects, political extremists, and subcultures≫. Since April 2020, Gab can be considered the most widespread alternative platform in Western countries, with twenty million users daily, born as Twitter and Facebook alternative social media. The alternative social media platforms are intended as other connection services between users, which is halfway between a social media and a discussion forum born to boycott the censorship actions of the main social media platforms (Meta Group, Twitter, etc.) and celebrate free speech even on controversial positions. How are sensitive topics, such as the one that concerns the skepticism related to the approvals of vaccines during the pandemic, addressed on the alternative social media platform compared to how they are dealt with on the mainstream social media platforms? This explorative work wonders about the users' points of view on vaccine concerns and the relevant differences between Gab and Facebook in addressing this topic. The empirical part of this work has been set starting from the dataset composed of Gab and Facebook content posted between March 2020 and July 2021. The posts were extracted with web scraping techniques (for Gab) and proprietary data tools (for Facebook), querying the keywords: *vaccine, vaccines, anti-vax (no-vax), Covid, Covid-19, coronavirus*. The collection procedure considered the different platforms' structure and their different organization of the interaction spaces. The population consisted of 8000 English writers' posts, from which 2000 posts with the highest interaction value were extracted. The dataset was analyzed using Topic Modeling, Factor, and Classification Analysis techniques. Our work's methodological output deals with comparing these social media platforms, bearing in mind their ontological objects and their algorithms' role. From the analysis emerged the differences and similarities of the social media platforms in terms of the type of content published, rates of involvement, sources of information, and directions of the considered speech. These differences have been duly highlighted by three clusters related to discourse orientation and communication approach: Conflict of views, Emotional externalization, Recommendation and practices. In addition to the type of communication and information circulating on a powerful platform such as Gab, the results help us understand the different narratives promoted on the two social media platforms and their role in the possible promotion of the same sentiment, opinions, and ideological polarization.

## Introduction

Digital cultures play an essential role in radicalization processes and are always considered a relational experience (online or offline), with a significant impact on consolidating individual and group identity (Antonelli, 2022).

According to Dow et al. (2021), the pandemic has disrupted traditional and social structures to which we were accustomed. Facing these disruptions, people ≪turn online to seek alternative cognitive and social structures. Once there, social media radicalizes beliefs and increase contagion (rapid spread) and stickiness (resistance to change) of conspiracy theories≫ (Dow et al., 2021, p. 1).

In this way, increased online presence during the COVID-19 pandemic and the long periods of lockdown have created a particularly fertile ground for spreading conspiracy theories, especially of the right-wing (Bessi et al., 2015; Antonelli, 2022). The spread of conspiracy theories is a particularly crucial problem today given the impact such beliefs have on individual behavior and, therefore, collective behavior, for instance, the people who believed the pandemic was all a farce and decided not to adopt the preventive behaviors recommended by the government (Chan et al., 2021). It is even more so if we consider the ease with which these theories generate radicalization phenomena that can lead to forms of polarization and violent extremism. The power of mass media in social and political changes is not new (Thompson, 2011); the real revolution is the speed at which social media platforms respond to users' social connections and information needs worldwide. In the end, the permanence of conspiracy theories and the subsequent radicalization process is facilitated by the way platforms work based on algorithms that facilitate the circulation of information in line with users' opinions (Cinelli et al., 2021). In this way, following Terren and Borge-Bravo (2021) and Del Vicario et al. (2017), if a user tends to consume conspiratorial content on social media, then the platform will tend to offer him more content in line with his conspiratorial interest to optimize his user experience. Consequently, the platform will indirectly reinforce his opinion and not allow him to come into contact with content that may question his conspiratorial opinion.

Ascertaining this background, this work aims to investigate how content relating to the Covid-19 pandemic and the vaccination campaign are communicated on alternative social media platforms, precisely because of their different regulation than traditional platforms. These are used to host content and content creators that traditional social media platforms may not tolerate.

At the same time, many studies have discussed the role of traditional social media in disseminating alternative information related to COVID-19 and vaccines (Chan et al., 2020; Gesser-Edelsburg, 2021). On the one hand, it is possible to hypothesize that alternative social media platforms can become attractive to conspiracy theorists and other users marked by radical positions already banned by traditional social media platforms; on the other hand, they are also frequented by users who have intentionally migrated from other digital spaces not attended by institutional actors and where content regulation policies are not (or differently) addressed (Innes and Innes, 2021).

The purpose of this work is not oriented to understanding which conditions push banned users from migrating to alternative social media platforms. Instead, it is to shed light, by a first comparison exploration, on the many ways to approach such a relevant topic surfing different regulated digital spaces. Gab and Facebook are two social media platforms offering different interaction modes and functionality (Cinelli et al., 2020a). The first one has been chosen for this case-comparison study because it can be considered the most widespread alternative social media platform in Western countries with twenty million users a day (Longo, 2021) born as Twitter and Facebook alternative social media (Nieborg and Poell, 2018). The alternative social platforms are intended as other connection services between users, which is halfway between a social media and a discussion forum born to boycott the censorship actions of the main social media platforms (Meta Group, Twitter, etc.) and celebrate free speech (Zannettou et al., 2018).

Although Gab and similar social media platforms may have been created and populated by users with explicitly political aims and discourses, their consolidation and growing popularity have inevitably led to other non-political topics being discussed online (Dehghan and Nagappa, 2022). In particular, the vaccine topic (Broniatowski et al., 2021). Facebook, on the other hand, can be considered the most established and popular social network site that enables relationships and discourse patterns between digital publics. Such patterns are intended as social formations made of temporary associations and cooperations among strangers with mutual agendas which disappear after a few hours of intense shared experience (Arvidsson and Caliandro, 2016). Our goal is to explore the narratives on them. In light of this, the research questions that motivate the exploration of the present work are:

- RQ1: What narratives are being built around the themes of COVID and vaccination campaigns on Facebook and Gab?
- RQ2: Considering the different functioning of the alternative social media platform, do users radicalize about vaccines and COVID issues?

## From alternative to extreme social media platforms

Social media represents an essential space for individual self-expression and collective association; however, users often transform the freedom these social media platforms offer to promote hate speech, disinformation, and conspiracy theories. To improve the safety of their users, social media platforms enforce their terms of service by performing strict moderation that includes removing certain contents or suspending particular users.

These actions have in turn led to the emergence of a substantial “alternative ecosystem” (Rogers, 2020), i.e., a set of discussion platforms (microblogging, social media, and messaging services) of less regulation and moderation that are used to host content and content creators that are not tolerated by traditional social media platforms (like Twitter and Meta group). What is “alternative social media”? How can we distinguish it from “traditional” social media? To answer these questions, Gehl (2015) conceptualizes alternative social media platforms using the classic alternative media theory that arose before the dominance of social media giants such as Facebook and Twitter. Alternative media were born as a response to older mass media and to counter its large corporate power to promote communication and the construction of public opinion along democratic lines.

This definition makes it difficult to consider “traditional” social media as an alternative to mainstream media. On the one hand, the dominant social media – Facebook and Twitter – may be seen as a first response to the public's demand for broader participation in the production and distribution of content. On the other hand, however, it cannot be denied that these platforms have maintained or even intensified some of the characteristics of mainstream mass media power described by earlier alternative media theorists, such as centralized and controlled communication (Gehl, 2015).

Indeed, traditional social media, being proprietary profit-seeking companies, can become hostile to ideas, discourses, and organizations, especially when in conflict with corporate hegemony, while allowing people to be content producers. So, as Gehl (2015) claims, alternative social media can be seen as a critical response to traditional social media that allows users to share content and connect and have more access to shape the underlying technical infrastructure and radically experiment with surveillance regimes.

Indeed, these social media platforms promote themselves as alternative solutions for those who want a different social media experience or are dissatisfied with the content moderation of major social media platforms. Over time, this business logic has supplanted the idealism of a free and participatory space that was promoted when the first social media was born, just as censorship and the algorithmic manipulation of messages have replaced unlimited social flows (Poell, 2014).

The result of all this has been the birth of alternative social media platforms that try to recover the founding principles of the first social media. This demonstrates what Waltz (2005) and Atton (2002) argue: there will always be alternative media alongside traditional media. Therefore, dissenting opinions and practices will always find new spaces of expression in opposition to the hegemonic ones.

While on the one hand, alternative social networks were created to offer decentralized and accessible methods of content production that challenge the power of proprietary companies, on the other hand, this free content production can take on dangerous characteristics. The infodemic (Rothkopf, 2003) understood as facts, mixed with fear, speculation, and rumor, amplified and relayed swiftly worldwide by modern information technologies has contributed to the out-of-control spread of conspiracy theories and disinformation.

Recently, the infodemic effect has been made acute by the COVID-19 pandemic in the way people were exposed to large quantities of both accurate and misleading information related to a health topic (Buchanan, 2020; Eysenbach, 2020), trying to ‘know what or whom to trust, especially when faced with conflicting claims or information' (Gruzd et al., 2021, p. 2).

To address the issue and respond to growing public and regulatory pressure, traditional social media platforms banned all conspiracy theory-related content, with influential conspiracy theory personalities and controversial entities and groups restricted or prevented from spreading extreme narratives (Mahl et al., 2021).

In this way, traditional social media platforms increased their role as content moderators and expelled users or groups that promoted controversial content from their services. Rather than limiting the circulation of this type of viewpoint, however, these actions have led to the emergence of alternative social media platforms that soon became a comfortable refuge for conspiracy theorists and other users with radical positions, obscured by traditional social media platforms which were not seen as a suitable communication space to express their positions. In addition to defining them as “alternative” social media platforms, we could also define these discussion spaces as “extreme” social media platforms, understood as those platforms on which users with extreme positions deplatformed by traditional social media platforms gather to share their radical ideas freely.

The emergence of these hyper-radicalized communication spaces motivated the genesis of our work. It is possible to argue that extreme social media platforms share communication practices with traditional social media platforms (the users can create, share, and interact with content in different ways), the essential differences being the organization of the interaction spaces and the criteria for selection of content on the platforms (Cinelli et al., 2020a). Following Zeng and Schäfer (2021) in highlighting the characteristics that differentiate extreme social media platforms from traditional ones, we adopt the theoretical framework proposed by Nieborg and Poell (2018) which includes governance, users, and technological infrastructure.

In regard to governance, these social media platforms celebrate the liberation of content: they have gained popularity by promoting their image as defenders of freedom of information, often resulting in conspiracy theories, racist hate speech, and toxic information being promoted (Thibault, 2016). These alternative social media platforms thus host personalities with radicalized and controversial positions who no longer find freedom of discussion on traditional social media platforms that moderate content. The lack of acceptance by society (or in this case by the digital space) is an additional emergent item of the radicalization process (Antonelli, 2022).

This perception leads users to seek refuge, acceptance, and understanding within digitally created ad hoc spaces dominated by extremist, simplistic narratives based on the exaltation of violent positions.

Regarding the technological infrastructure and the functioning of alternative extreme social media platforms like Gab, no apps are synchronized because they are banned from all app stores. It differs from social media platforms like Facebook, which depend heavily on the news feed algorithm (Acampa, 2022). In this way, on an alternative extreme social media platform such as Gab, content producers can communicate with, and receive suggestions, directly from their most loyal followers who support their positions.

This migration can be considered as the full realization of a selective information space: in this case, the user gets out of the echo chambers of traditional social media and immerse himself in a real “echo platform”, i.e., platforms in which the contents produced and shared are exclusively in line with the beliefs of the users who live in it.

While many studies have questioned the role of traditional social media in disseminating “alternative information” (Cinelli et al., 2020b; Jhaver et al., 2021), these alternative social media platforms remain little investigated in social media studies. For this reason, it is interesting to understand how these social media platforms operate in the new peripheries of the web (Thibault, 2016) and form a part of a broader communication ecology.

## The role of social media in the COVID-19 pandemic

The use of the web and social media during the first phase of the pandemic significantly increased, allowing users to stay connected with friends, relatives, or colleagues, disseminate protocols on care and personal protective equipment, and retrieve real-time information about what was happening at that moment. Social media played an essential role in creating a sense of community and solidarity during lockdowns and restrictions imposed by the pandemic. Social media users shared individual experiences and stories of daily life, creating a sense of belonging and closeness among people facing similar problems (Wong et al., 2021).

According to data produced by “We Are Social” in January 2022, out of a global population of 7.91 billion people, there are ~5.31 billion mobile users (67%), 4.95 billion users connected to the Internet (62%), and 4.62 billion active people (which generally means a person who has connected at least once a month) on at least one social media (58%). These numbers describe a digital transformation underway for almost two decades globally as Internet and social media penetration rates have constantly increased. The growth of users of social media platforms has more than tripled in the last 10 years, from 1.48 billion in 2012 to 4.62 billion in January 2022, which is an annual average growth of 12%.

Since the unexpected beginning and first wave of the COVID-19 pandemic, global terms such as social distancing, gathering, smart working, and lockdown have assumed importance. Social media platforms have proved to be potent tools as they have allowed all users, especially the most followed, to spread the rules to limit the spread of the virus as much as possible.

Among the most characteristic functions of social media platforms during this pandemic have been the rapid dissemination of protocols at regional, national, and international levels. Sharing protocols about treatment, personal protection equipment, or even proposals for fair allocation of scarce resources in medical settings have now become the new standard (González-Padilla and Tortolero-Blanco, 2020).

They played an essential role in communication during the pandemic, providing a channel through which people could stay in touch and exchange information about the spread of the virus, prevention measures, and safety recommendations (Wong et al., 2021).

However, as the manufacturing and distribution of vaccines ramped up, false and misleading information about vaccines' efficacy, safety, and side effects increased on social media (Gruzd et al., 2021).

On the other side, subjective opinions, prejudices, and conspiracies have been intertwined with these reports, generating an enormous proliferation of false news and misinformation in general. This has also occurred because of the speed with which the news circulated, also among the main newspapers.

Of course, when we think about social media platforms, one cannot ignore what Pariser (2011) called “bubble filters”, according to which users live in a “custom ecosystem” governed by algorithms that choose and predict preferences and results according to their own biases. These bubbles produce a cycle of comparable content that prevents the user from seeing other sources to counter the information and, therefore, any fake content. The use of “social media platforms was perceived as easy and accessible to everyone for sharing, posting, and reacting to any information – also medical information – regarding the pandemic. While people continued to work from home and ensured social distancing, most users supported family and friends and attempted to raise awareness by sharing and circulating a range of information within their closed social networks (Saud et al., 2020).

However, misperceptions negatively influenced the perceived severity, susceptibility, and efficacy of government preventive measures, which may have ultimately resulted in decreased compliance (Meppelink et al., 2022).

During the pandemic, social networks were the place for four types of users to meet and clash: pros, cons, neutrals, and those hesitant about the scientific aspects of the vaccination campaign. In this case, social media platforms, particularly those with large user bases, were most culpable of spreading vaccine-related misinformation that may have contributed to vaccine hesitancy (Gruzd et al., 2021). Several times in this way, social media contents are featured by verbal aggressions that can be framed as “a personality trait that predisposes persons to attack the self-concepts of other people instead of, or in addition to, their positions on topics of communication” (Infante and Wigley, 1986: 61).

Many studies described how social media, despite their efforts to remove COVID-19 vaccine–related misinformation, have featured as vectors for vaccination hesitation in many countries worldwide (Lou and Ahmed, 2019; Burki, 2020; Gruzd et al., 2023). In this way, hesitant reactions were driven by many different reasons related to any other effects of misinformation as in the AstraZeneca case in Italy (Crescentini and Padricelli, 2023), through false crossclaims about the vaccine efficiency ‘not constrained within a single platform' (Gruzd et al., 2023). Considering this, public communication policies provided fast resilience policies oriented to monitoring and restraining the impact of misinformation, taking care that as in the U.K. case, 'government-generated messages may be less effective than those of other actors' (Bloomfield et al., 2021)

According to this, social media platforms have had the role of disseminating not only scientific and technological content but also the opinions of scientific experts even though they are not widely used. The importance of scientific communication – understood not as “popularization”, that is, bringing people closer to knowledge but as a means to transmit and spread messages – is constantly neglected and denied by scientists. However, there are many exceptions in the national and international scene. In this way, scientific experts struggled to maintain their previously unchallenged authority due to differing opinions and inefficient communication. At the same time, the web broke the sequential order and tightness of a series of 'filters' that previously distinguished the pathway of scientific results from the researcher to the general public (Bucchi, 2006, p. 72).

## Alternative social media platforms and alternative narrations?

To answer the research questions mentioned in the introduction section, this work, from a comparative perspective, will explore the narratives of two examples of these respective types of social media platforms, Facebook and Gab, as they offer different modes of interaction and the ability to amplify content. It will also reflect on the ontological aspects of the social media platforms themselves, such as the role of algorithms in the dissemination of content and the polarization of public opinion. The data were collected through a query strategy and then the database was subjected to a content analysis. To better answer these questions, an in-depth comparison was made of content posted by users on Gab and Facebook between March 2020 and July 2021. The data collection procedure involved gathering content posted by users on both social media platforms in the selected timespan through a query that contained the following keys: vaccine, vaccines, anti-vax, COVID, COVID-19, coronavirus. Web scraping procedures were used for Gab's extraction while for Facebook, we availed ourselves of Crowdtangle, an insight tool reserved for the academic community that only tracks publicly available posts on Meta's group social media platforms.

To achieve the objectives of this research, it is necessary to equip oneself with the tools and techniques useful for extracting meaning from the available information. This is the case with content analysis, a technique aimed at breaking down any type of message into “simpler constituent elements, the recurrence of which can be calculated, also in view of further processing, possibly after classification in appropriate systems of categories” (Amaturo and Punziano, 2013, p. 24).

In this specific application case, which follows a content analysis of the third type (Rositi, 1988),^1^ the empirical basis was created by preparing a standardized grid for data collection (Table 1). Through this grid, the collection of all cultural products containing the selected keywords during the identified observation period was organized.

**Table 1:** Content analysis standard grid.

**Social media platform**	**Total reactions**	**External sources**	**Post type**	**Message**
Facebook - Gab	Low – Medium – High levels	Academic journal – Academic press – Amazon – Blogs – Cloud – Documents – Institutional – Petition – Scientific reports – Telegram – Twitter – Video Platform - Youtube	Link – Live video complete – Meme – Native video – Photo – Post – Screen – Status - Video	Post text

Thanks to the guidance provided by the grid, in addition to the identifying variable of the social media platform on which the collected content was posted, the variable regarding the total reactions to the posts made by users was operationalized. The latter consists of the sum of the number of likes, comments, and content shares. This cardinal variable was subsequently discretized into an ordered-categorical variable, which follows a specific ordering as an ordered series along a continuum characterized by a precise range. This transformation operation was conducted by identifying the central value along the frequency distribution of the entire variable and then dividing it into tertials, where each interval had an equal number of observed cases. Subsequently, the variable was reclassified into low-medium and high reactivity levels, respecting a monotonic relationship between the intervals (Marradi, 2007).

The empirical base initially composed of 8000 observations was reduced to a dataset of 2000 posts, selecting an equal number of cases retrieved from the two social media platforms. The selection of the first 1000 posts from Gab and the first 1000 posts on Facebook was based on replies to rates (we selected 333 low-rate, 333 medium-rate, and 334 high-rate posts from each platform).

Furthermore, variables related to the type of published post and the nature of any external sources contained within the published material were operationalized.

The first variable consists of identifying the communicative codes characterizing each case observed, whether they are textual (post and status), audio-visual (live video complete, native video, photo, screen, meme, and Video), or contain hyperlinks to external social media platforms (Link).

The origin of these external sources is operationalized in the second variable, which distinguishes those contents not belonging to Gab or Facebook. Among these, there are academic-scientific sources (academic journals, academic press, and scientific reports), commercial sources (Amazon), blogs, document archives (cloud and documents), institutional press releases, fundraising campaigns, petitions, and other social media platforms or instant messaging platforms (YouTube, Twitter, Telegram, and Video platform).

All the collected data were processed with a multi-stage content analysis that consisted of three separate analytical phases: the application of the main seven topics of *topic modeling*^2^ from our dataset in order to understand the narrative features created by users among Gab and Facebook; the application of a *Lexical Correspondence Analysis* (LCA)^3^ to detect the latent mining dimension detectable from the combination of the emerging topics with the proper functions of both social media platforms; and a *cluster analysis*^4^ that synthesizes all of the information used in few and homogeneous groups.

## The topic model and LCA

After building the dataset, the first analytical procedure was used to identify, via the considerable amount of information in the textual variables, the narrative features related to and emerging from the material collected. Despite the difficulty in tracing the semantic structure of the texts, topic modeling offered an empirical basis for a simple, automated, and statistically robust solution. This technique identified topics within the analyzed works that were explored through the textual variable. As the first step, the database was imported into T-Lab, a specific quantitative content analysis software that processed patterns based on textual context. Later, the textual variable was submitted for the thematic analysis procedure of T-Lab. Prior to the preparation of the text for automatic analysis, lemmatization, lexicalization, and segmentation procedures were followed and finally, the frequency threshold was set at 10 occurrences. In this way, a final database comprising ~969 total words was obtained.

After this corpus building, a model was set up through which seven topics were extracted and appropriately renamed to respect statistical criteria as follows: the consideration of specific word occurrences featuring the topic; low-high shared word occurrences among all topics; and semantic tagging of selected contexts in order to “detect the right document meaning in order to solve disambiguation and identifying concepts by a set of words.” (Bolasco, 2013, p. 126). Since each of the texts analyzed comprised a document composed of words, this text could be subdivided into elementary contexts, i.e., smaller portions of text such as sentences, in which the specific combination of words was more or less likely to lead back to a specific topic. The topic would then be the set of specific words belonging only to that topic plus several other words shared with greater or less probability with other topics. The combination of these sets of specific and shared words leads to different semantic universes. This allows the topic to be correctly labeled considering the specific words, shared words, and elementary context l within the topic, which helps contextualize the words in specific portions and deduce a general meaning of the topic.

Finally, as summarized in Table 2, the seven topics were classified, taking account of the 3,450 emerging elementary contexts that comprise the total corpus of documents analyzed. Following Habert (2005), the greater significance of parts of the documents depends on the information weight of its elementary contexts, featured by its discursive formulas, their position in the document, and the specific weight of each word related to its distribution in the document. In this study, T-Lab returned the following summary of elementary contexts with a hierarchical order based on the informative score of single elementary contexts in which text reduction has been synthesized by a 95% threshold.

**Table 2:** Topic name, featuring words, and examples of elementary context.

**Topic name**	**Featuring words**	**Examples of elementary context**
Business Spamming	business; need; support; help; service; learn; work; job; money; time	−5 Days To Go! Are you pivoting toward a pandemic-proof business? Get to showcase what you do or your business to The Business_Marketplace_Global community through your 60-sec pitch. Find solutions to your business needs by meeting people with the right skills and knowledge. Meet new prospects and generate referrals to get high-quality leads.
Corona updates and behavioral communication	active; IndiaFightsCorona; case; Texas; Unite2FightCorona; restaurant; recovery; StaySafe; Total_Cases_till; Total_Recoveries_till total_tests_till; sanitize	- # IndiaFightsCorona # Unite2FightCorona # Maharashtra # COVID19_Updates as_of 18/06/2021: and New Cases - 9,798 andRecoveries - 14,347 andActive Cases - 1, 34, 747 and Total_Cases_till date - 59, 54, 508 and Total_Recoveries_till date - 56, 99, 983 and Total_tests_till date - 3, 90, 78, 541 # StaySafe # Unite2FightCorona # IndiaFightsCorona
Home business speculating	fee; stayhome; ppe; workfromhome; facemask Coronavirus; workathome quarantine; onlinejobs homebusiness howtomakemoney internetmarketing makethatmoney	- Get Free “Viral” Traffic For ANY Website or Affiliate Link, With 1000s of REAL Visitors. In Just 41 Seconds! #howtomakemoney #makethatmoney #workathome #workfromhome #homebusiness #internetmarketing #onlinejobs #coronavirus #lockdown #stayhome #pandemic #quarantine #facemask #ppe #KN95 #N95 #Covid19 #stayathome
Quarantine diary	COVID19; love; day; god: view; music; hope; play; window; howl; enjoy; stay safe; quarantine beautiful; home; kind; stay; fire; walk	- Day 22 of #lockdown. Pretoria, South Africa ZA. A big Highveld thunderstorm is brewing after a chilly day. Rolling thunder and lighting. Home is the best place to be right now. Stay safe #Covid19 - Staying home in Indianapolis, IN, USA, sitting on my couch, looking at the window, and thinking how fortunate I am. Stay home, Stay safe! Photo taken April 26, 2020 #Covid19
Risk perception and Covid treatments	virus; study; cause; cell; risk disease; vaccine; university; spike protein; ivermectin COVID-19; approve; SARS-CoV-2 rate	- One of the basic conditions for the emergency use authorization granted to the vaccines currently being used against Covid is that there are no alternative treatments available for the disease. As such, if ivermectin or some other promising medicine such as fluvoxamine were approved as an effective early treatment for Covid-19, the vaccines could be stripped of authorization. - That might explain the company's recent statement claiming that there is “no scientific basis whatsoever for a potential therapeutic effect of ivermectin against COVID-19. If approved as a Covid-19 treatment, ivermectin could even threaten the emergency use authorization granted to Covid-19 vaccines.
Skepticism and critics of pandemic	people; patient; vaccinate; doctor; mention; Fauci; vaccine; tell; speak; government individual; inject	- Knowledgeable, informed Doctors around the world are warning people to stay away from those vaccinated. Vaccinated people are now believed to be super-spreaders who can shed through their skin, pores, and breath to those not yet vaccinated resulting in those people being infected as well.
Vaccine effectiveness	vaccine; death; die; COVID mention; ellipsis; REPORT; vaccination; invisible; Gate Case; data; COVID_VACCINE Mrna; Vax; reaction	- For Second Week in a Row: More COVID-19 Vaccination Deaths than COVID-19 Deaths in the US according to CDC and VAERS Websites - - by Jim Hoft. There are now 11,140 reported deaths from the COVID_VACCINE in the United States. This is up from the 9,125 reported deaths from the COVID-19 vaccinations total from last week. Two weeks ago VAERS reported 6,985 deaths due to the COVID vaccines.

**Figure 1:**
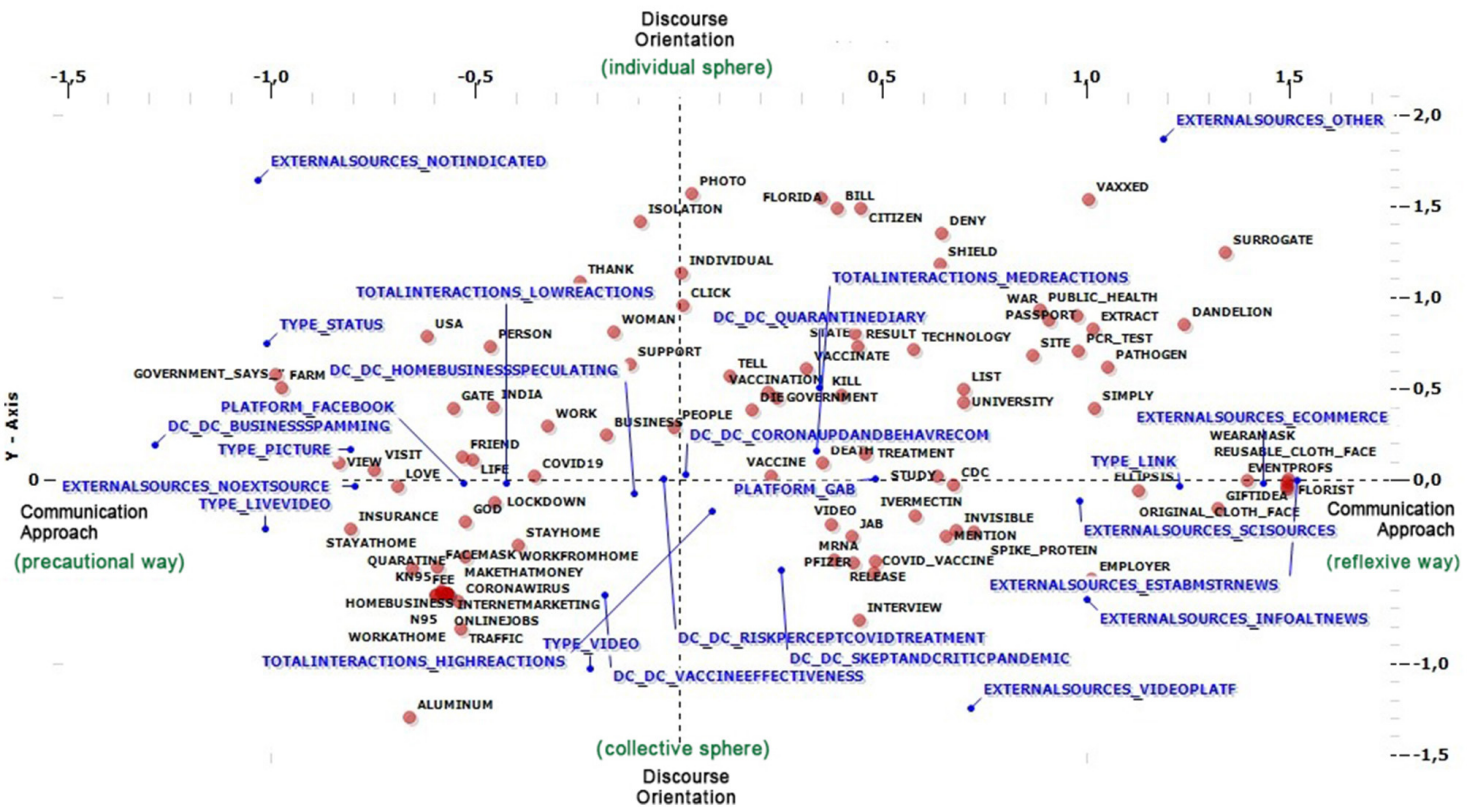
Lexical correspondence analysis.

**Figure 2:**
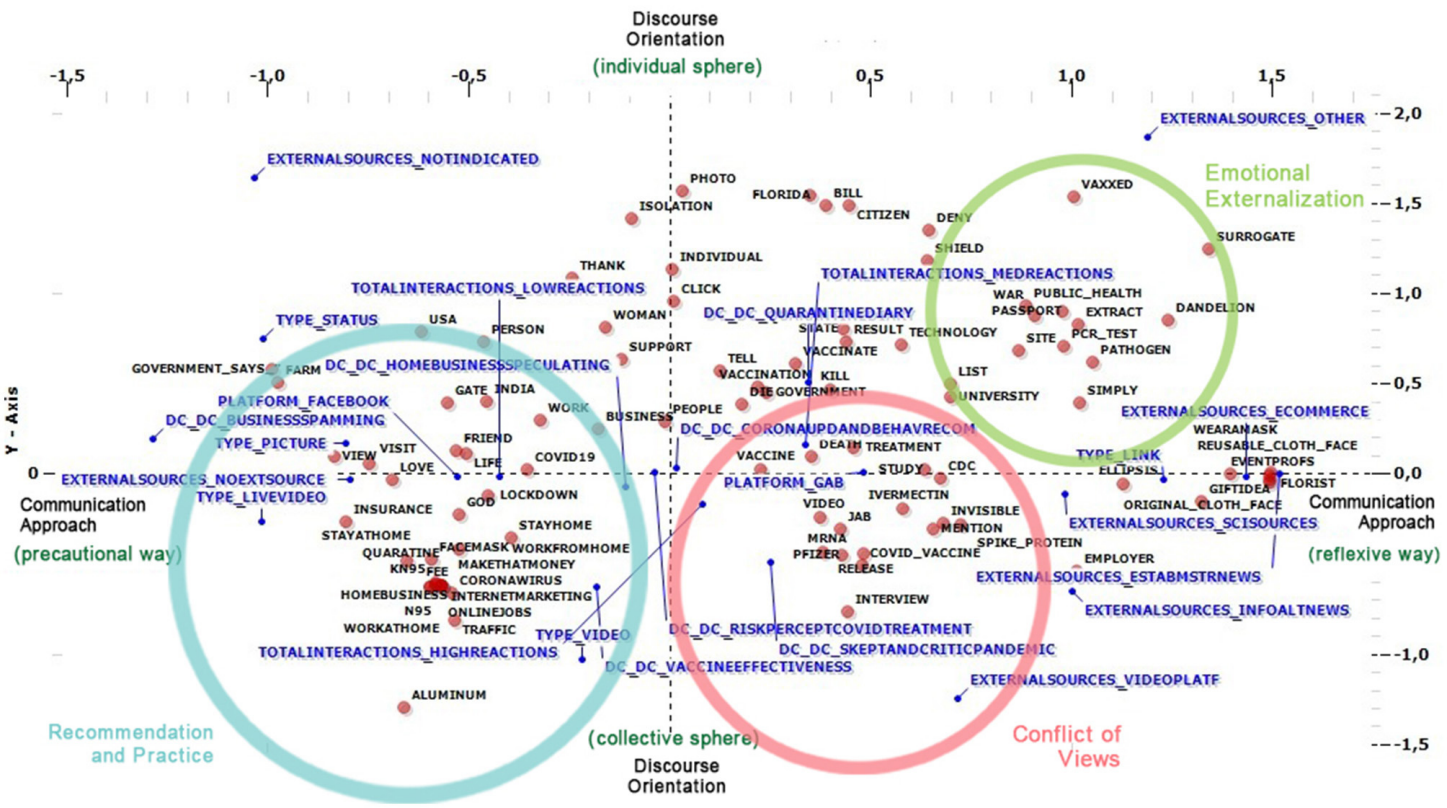
Cluster analysis.

Lexical Correspondence Analysis (LCA) was then implemented to answer RQ1 to encapsulate the narration on both social media platforms, Gab and Facebook, and underline the main differences between them in framing and discourse-building about the COVID vaccine issues during the pandemic period. After this, a cluster analysis was implemented to retrieve three uniform groups representing the major communication patterns users utilize. Figures 1, 2 show the main results.

Before discussing the results of the analysis, it is necessary to describe how the LCA (Lexical correspondence Analysis) plan is constructed and how emerged the main latent dimensions thanks to the multidimensional approach we used to process and analyze textual material. The first one, on the horizontal axis, is concerned with the primary communication approach to Covid and vaccine issues. It synthesizes the reactions to the pandemic situation during the first period of the pandemic. Facebook and its related behavioral approaches are to the left of the axis. Here, we emphasized and concentrated on the precautionary ways of self-protection from contagion, such as staying at home and avoiding interactions. Contrariwise, the right of the axis where Gab is positioned focuses on the meditative and wise approaches open to the debate among users and their reactions to the pandemic issue over the longer term. Here, it is possible to detect those posts linked to the discussions on the control measures, COVID victims, and vaccine reliability.

Along the dimension shown on the vertical axis, higher and lower values relate to discourse orientation characterized by individual and collective experiences, respectively.

On these bases, the narration on both social media platforms is concerned with four discussion frames that emerge across these dimensions. The first frame, which emerges in the plan's first quadrant, is characterized by a sensible approach to the pandemic issue. This is oriented on the micro level and by the direct individual perspective of users. The topic related to the quarantine diaries here gathered all those elements related to the individual narration of daily life during the pandemic. This narration practice makes sharing the experiences held during the lockdown possible. It is also supported by the contents related to the contagion updates and the first treatment developed to fight the virus. In this way, a narrative reflecting conventional or popular wisdom is more concentrated than the more scientific-oriented ones focused on in the fourth quadrant, considering the modality of scientific sources (Academical Journals, Academic Press, and Scientific Reports) from the variable of external sources addressed.

Furthermore, thanks to the support of the most frequent words, such as Pfizer, mRNA, spike protein, release, and Covid vaccine, this quadrant concentrates on all those contents that deal with more detailed information concerning the pandemic in general and the vaccine issue. In this way, a narration is featured by commenting and reviewing discussions of the scientific sources addressed by users. Among the latter, scientific papers have also been included in the preparation of the data-gathering procedures during the operativization phase.

The second quadrant takes shape as the more speculative one. Here, we find a relative need for more discussion among users. There is instead more space reserved for storytelling practices that distinctly emphasize the potential fears the socio-economic insecurity can cause because of the lockdown isolation, a fertile ground for questionable business practices. Lastly, in the third quadrant, which crosses the impact reaction and the collective sphere, it is possible to find the narrative refrain of “stay at home” and the other behavioral practices that individuals adopted during the first pandemic phase. In summary, looking at the graph, the narration of Gab is featured in users' confrontations and debates on a critical approach to the pandemic.

Moreover, despite the primary identifications of users present on Gab, who are defined as radicalized by scholars (Lima et al., 2018), no rude communication styles emerge from the empirical evidence in this case. At the same time, the LCA reveals a proactive debate based on scientific sources if a ≪polarized perspective of user opinions, bubbles, or any other elements linked to the reinforcement of their political or social views≫ (ivi) is not detectable. Moreover, despite what was initially expected from Gab, no opportunistic practices or phishing post-trends are disseminated by the self-regulated users, unlike what emerges when looking at the part of the graph in which Facebook narration is framed. On the one hand, the interpretative schema built on the popular social media platform appears shaped for a digital space to report “what's going on”, in other words, to comprehend the contagion trends and to be informed about the pandemic issue. On the other hand, the debate about the situation related to the vaccination campaign goes into the background. It allows space for spamming and speculating content with no linked external sources that are not violating the strict company regulation.

The results of the later analytical procedure align with this vision. Later in the LCA, a further cluster analysis is provided. Finally, the synthesis operation is carried out on the plane constituted by the intersection of the two syntheses. In this way, it is also possible to characterize the narrative differences between Gab and Facebook thanks to the contributions provided to the topics surveyed, the social media platform's positions on the plane, and the position of three emerging groups.

As shown in Figure 2, the first group, which contains most of the information of the dataset (65%), gathers the social narration that was held on Facebook and was related to the collective sphere. Here, there are concentrated words and posts that reflect the recommendation and practices to be followed during the lockdown, which was experienced as the “stay at home” wave and the need to respect social distancing. As shown by the most frequent words inside this cluster, these recommendations gained more frequency and relevance than contents related to the topic of home business speculation in the cluster. The advice on approaching new forms of sociality, such as going outside only for emergency purposes, wearing masks, and promoting social distancing, occurred because of the pandemic. These recommendation practices have been replaced by those contents that concern and promote remote working practices from home that, as promised by the users, drive easy online economic earnings, such as online investments, buying and selling products, etc. In an opposed position, Gab takes precedence in the second cluster, comprising 25% of the information contained in the dataset, which relates to the collective sphere. In this case, elements concerning risk perception and discussion concerning vaccine effectiveness gain more space. Users' knowledge construction opened to the conflict of views between them and any other actor related to the Government or conspiracy engagements. As shown in Table 2, the elementary context and the more frequent words underline these conflictual oppositions: “Vaccinated people are now believed to be super-spreaders who can shed through their skin, pores, and breath to those not yet vaccinated, resulting in those people being infected as well”. In this last group is found content more frequently posted on Gab than on Facebook, constituting 10% of the full information. Here, content relates to the emotional externalization of the individual experiences during the pandemic. These are related to those social constructions that feature positive and negative experiences. They depend on the learning processes and the social mechanism connected to a new experience as the pandemic. Despite the lack of proper methods and instruments to detect and comprehend these elements, other investigative perspectives must support the study of emotions. In this case, the application of traditional techniques related to the qualitative approach can be helpful in two ways: to shed light on the traces already left reported by the quarantine diaries and then to identify interesting research paths to go in-depth into a specific emotion or sensation that can be later translated by individuals in a specific attitude, social construction, or action.

## Conclusion and future work

The content analysis highlighted the differences and similarities of the social media platforms in terms of the type of content published, rates of involvement, sources of information, and directions of the considered speech. The results helped to answer RQ1 and provided insight into the different narratives promoted on the two social media platforms. In fact, to the issue investigated and in contrast to what was hypothesized about the Gab social media platform, a particularly radical, sectarian, or conspiratorial environment did not emerge. The empirical exploration to compare Gab and Facebook underlines that Gab is a narration more oriented to structures, reactions, debating, and emotional assumptions, while with respect to Facebook, a more impactful orientation. Referring to RQ2, while Facebook functioned as the platform where users spread best practices to deal with the pandemic, Gab on the other hand emerged as a platform characterized by different narratives opposing the mainstream narratives, which includes users who address, on one hand, critical and sensitive issues related to the emergency, and on the other hand conflictual and critical public argumentations. In the case of this study, the alternative ecosystem that was born from an attempt to find spaces of expression not subject to regulation and censorship did not generate an information environment based on all the same information and positions. On Gab, different positions and consumption of different information sources were detected; this information source was curated collaboratively and used to formulate or reframe explanations about the pandemic and the vaccine campaign. This explains that extremism, in all its forms, is a cumulative and incremental phenomenon or a process that arises or amplifies as a reaction to exposure or prolonged contact with an ideologically different type (Antonelli, 2022).

The major focus of studies on social media platforms like Gab has limited itself to either explicitly political topics or certain problematic discourses and content such as hate speech, misinformation, and conspiracy theories (Dehghan and Nagappa, 2022), but our analysis shows the emergence of Gab as a space for comparison and sharing of information sources and differing worldviews that is lacking on Facebook. Gab claims to promote freedom of speech and provide an environment where users can express their opinions without fear of being censored or banned. Relating to RQ2, as LCA outcomes show, the themes investigated a user who expresses a position against the tide on Facebook that most likely triggers a real “shitstorm” against him, generating a polarization of positions that in a short time turns into hate speech, thus preventing a meaningful contest between different opinions. On the contrary, on Gab, the idea of being able to express a position (albeit controversial and not widely shared by public opinion) with a users' audience, who probably share that same position and are more prone to confrontation than to attack, generates a discussion environment that is less polarized and more open to the exchange of opinions. The lack of radicalization of communication may be associated with the sharing of opinions regarding particular themes and the non-existence of users who can counter them. A limitation of the work is undoubtedly linked to the themes investigated in a particular and singular historical moment for the entire world; different results could emerge if investigating incredibly divisive themes traditionally used to support conspiratorial propaganda. An expansion of the work could be done from a comparative perspective between social media platforms and relevant themes in each historical moment.

## Data availability statement

The raw data supporting the conclusions of this article will be made available by the authors, without undue reservation.

## Ethics statement

Ethical approval was not required for the study involving human data in accordance with the local legislation and institutional requirements. The research involving social media data was conducted in accordance with the platforms' terms of use and all relevant national and institutional guidelines.

## Author contributions

This article should be considered a collaboration, in particular, in its conclusions. However, the introduction and paragraph 1 are to be attributed to SA, paragraphs 2 and 3 to NC, and paragraph 4 to GMP. All authors contributed to the article and approved the submitted version.

## Footnotes

^1^Content analysis can be divided into ≪three different types based on the chosen classification units. In the third type, there is no decomposition into elements, but the classification unit coincides with the contextual unit. In practice, it is as if the examined communication were subjected to a real questionnaire, “asking” an advertising message, for example, if a female character appears, what role she plays, and so on≫ (Amaturo and Punziano, 2013, p. 55–56).

^2^A model applied to topic extraction was based on the Latent Dirichlet Allocation, a “generative probabilistic model for text document collections based on a three-level hierarchical Bayesian model, in which each item of a collection is modelled as a finite mixture over an underlying set of topics. Each topic is, in turn, modelled as an infinite mixture over an underlying set of topic probabilities. In the context of text modelling, the topic probabilities provide an explicit representation of a document” (Blei et al., 2003).

^3^Lexical Correspondence Analysis is a factorial technique concerning textual data and is useful to synthesize information contained in texts, make graphic displays of association networks among words and between words and texts, and show the connections between text and context data (Lebart et al., 1998).

^4^Cluster analysis is an analytical technique that aims to synthesize observed objects into a few representative groups (Lebart, 1994).
